# Working together for perioperative excellence in pediatric perioperative research

**DOI:** 10.1016/j.bjane.2026.844736

**Published:** 2026-02-05

**Authors:** Britta S. von Ungern-Sternberg, Aine Sommerfield

**Affiliations:** a Perth Children’s Hospital, Child and Adolescent Health Service, Department of Anaesthesia and Pain Medicine, Nedlands, Western Australia, Australia; b The University of Western Australia, Institute for Paediatric Perioperative Excellence, Anaesthesia and Pain Management Program, Perth, Western Australia, Australia; c The University of Western Australia, Division of Emergency Medicine, Anaesthesia and Pain Medicine, Perth, Western Australia, Australia; d The Kids Research Institute Australia, Perioperative Medicine Team, Nedlands, Western Australia, Australia

**Keywords:** Clinical excellence, Collaboration, Consumer, Multi-disciplinary, Outcomes, Perioperative Team

Pediatric perioperative care can be described as a journey, starting when surgery is first contemplated, all the way through to a patient’s full recovery. For the child and their family, this journey spans from the time at home pre-operatively through a hospital stay and finishes with at-home recovery. The continuum of care, ideally seamless, is extremely important to our patients and their families and requires close collaboration among all relevant clinical disciplines.

Pediatric perioperative research should follow the same path, bringing together experts from various disciplines to ensure that all medical, psychological, social, and functional considerations are taken into account as we work toward the common goal of improving outcomes and the perioperative experience for our young patients. Perioperative research collaborations can be thought of as a jigsaw puzzle, where the pieces must be assembled correctly so that we have the necessary skills to assess the problem from all angles, find solutions to our clinical questions, and guide innovation in the perioperative space. In some cases, this may involve partners from various institutions, locally, nationally and internationally.^1^^,^^2^

This jigsaw will be incomplete without the inclusion of consumers/patient/parent/community representatives/stakeholders (referred to as consumers hereafter for simplicity) to ensure that our research is truly patient- and family-centered. A diverse group of consumers should be involved in all stages of the research project, from conceptualization through to implementation and translation. Ensuring a diversity in any consumer involvement is critical. For example, the lived experience of children and young people will differ from adults who have lived perioperative experience as a child, coupled with their additional adult perspective, as well as from the experience of parents.^3^ Efforts must be made to engage with consumers from different walks of life, representing the breadth of our community, including minority groups and those from Culturally and Linguistically Diverse (CaLD) backgrounds, including First Nation representatives. Consumers can guide us not just on individual projects, but also on their research priorities, ensuring that the research we conduct is not only acceptable and useful to clinicians and researchers but also to our young patients and their families.^3^, ^4^, ^5^, ^6^, ^7^ Our research group has been purposefully involving consumers with lived experience in all of our research from bench to bedside and endeavors to involve consumers and community in the four stages outlined in the Guidelines from the Australian National Health and Medical Research Council:^6^ 1) Research prioritization; 2) Developing research concepts/hypotheses/questions and designing research projects; 3) Research conduct including participant recruitment, consent and responsibility (ethics, governance) and oversight or governance of the conduct of the research and 4) Reporting, communications and publication/dissemination and translation. In line with institutional and state practice, our consumers are reimbursed for their time. To date, we have not acknowledged consumers in all our research publications, but the decision has been taken to do this more systematically in the future.

While this seems like the obvious standard for patient/family-centred care and research, we wonder whether meaningful consumer involvement is as widespread as some believe. Additionally, meaningful consumer involvement is distinct from consumer consultation or participation in studies.^8^

We have received pushback from some interstate and international collaborators and manuscript reviewers who questioned the appropriateness of consumer involvement due to the consumers’ perceived lack of medical and subject-specific expertise. We have been asked how the consumer could know the details of the research? How and why should they know about something that happens when they are asleep? Patients and families have the right to participate in shared clinical decision-making and receive full information about their treatment, the same holds true for research. It is also reductive to consider the perioperative experience as only concerning the time under anesthesia. Additionally, a significant number of trials are funded by government agencies around the world. There is not only the need for full transparency on how the public funding is spent, but the public should also have their voice heard on which projects should be prioritized.

In addition to consumers, a multidisciplinary research team should not just reflect the multidisciplinary perioperative clinical team but also include, from the beginning, biostatisticians and data scientists. These are vital to ensure that research is designed in the appropriate way to test our hypotheses, that our sample size is sufficient and to build a solid data analysis plan at to ensure as early biostatistics/data-science involvement strengthens methodology and rigor. While this is standard practice in large centers, access to statistical support can be much harder outside large academic centers and in less resourced settings. This is critical to ensure that research is not only approved by an ethics committee but also performed in an ethical manner and will ensure that the study design can actually answer the research question.

We also need to ensure that any research outcomes are economically beneficial and implementable. This is an increasingly important imperative, given that the pockets of our healthcare systems are not bottomless, and society is increasingly confronting aging populations and rising healthcare needs. Mindful of the resources available to us as a community, we should assess the cost-effectiveness of proposed research interventions. Health economists and implementation scientists should be included, not as an afterthought, but from the conceptualization stage, to improve the translation and implementation of any findings.

Last but not at all least, research teams require the operational backbone, the clinical research coordinator or clinical program manager, the person who is responsible for the day-to-day running of the study. While this practice may be standard in large, well-funded academic institutions and, in fact, may form part of the requirements in some Ethical/IRB processes, it is not universally followed, mostly due to resource constraints. This role can span a range of functions, depending on the individual research team's set-up, investigator skills and time, and institutional/country norms. One function can be consenting participants for research, which avoids the potential for subtle coercion of patients into research participation, thereby reducing the perception that enrolment into the research is a requirement and also reducing the risk of therapeutic misconceptions.^9^

A major benefit of the research coordinator/research manager role is to take on administrative/managerial tasks that would otherwise fall to the clinical principal investigator, who is commonly the clinician team lead behind the project. In reality, the medical principal investigator is often busy, often wearing many hats, e.g., clinical care, research, and teaching. The research coordinator, on the other hand, can focus fully on protocol adherence and team communications, as well as managing timelines, reporting, and budgets, ensuring that all necessary information is collected and maintained to the highest standards. The research coordinator often leads the hub for a multidisciplinary team, ensuring seamless communication between all parties involved, including close communication with all participants and their families.

## Beyond simple teamwork

Collaboration within a perioperative, multidisciplinary research team is not as easy as assembling a group of experts. Such a group needs to learn how to work together as a cohesive team. Different craft groups may have different viewpoints; they attempt to set a different focus. This may set the scene for conflicting priorities within the research team. A clinician, for example, is likely to prioritize patient care and comfort, while a statistician pushes for a double-blinded study – potentially leading to discussions. If these differences are discussed constructively, it can be highly beneficial, leading to better mutual understanding, more innovative research protocols, improved study designs, and ultimately, better patient outcomes. However, if not led constructively and respectfully, these task conflicts have the potential to evolve into relationship conflicts, which are detrimental to any team. It is therefore vital for all team members to separate the content of the discussion or disagreement from a personal level. Different viewpoints should not be seen as a personal attack, but rather as an opportunity to benefit from a diverse team dynamic and work together as equals to forge a better path through the complex perioperative jungle.

If all parties work together collaboratively and respectfully, putting their egos aside, then the work will not only be more enjoyable but also more constructive. It may be a challenge for some clinicians, trained and experienced in the traditional medical hierarchical structure, to accept non-clinical staff as equals and adapt to a multidisciplinary dynamic, but this improved culture and collaboration will make any team thrive. Adding differing perspectives ensures that researchers are not in an echo chamber. It will further aid the successful implementation of the research findings, with all individual specialists working together, both clinically and in research, driving the clinical and research agendas in parallel, thereby impacting innovation and clinical change with a shared vision to improve patient outcomes.

For this to be possible, we need to learn each other’s language and develop a common language and understanding based on mutual respect for each and every partner in the team, recognizing their strengths and weaknesses. In larger projects, building a formal collaborative framework may be helpful, to define roles, objectives, a clear roadmap, expectations and contributions.^1^ From personal experience, our large multidisciplinary team, involving many clinical and non-clinical specialties as well as consumers of all ages, with a wide range of experience and skills, is what drives us, what motivates us; we keep learning from and with each other. It lets us grow individually and makes us stronger as a team. It allows us to design better projects and to drive our vision together to improve the safety, care, and outcomes for all children requiring perioperative care. Only together, we have the jigsaw pieces required to see the full picture. Only together, we can find solutions which improve care for all our patients around the globe.

## Actions to establish and sustain multidisciplinary pediatric perioperative research teams

• Ensure an appropriate team composition including clinical and non-clinical multidisciplinary expertise (e.g., medical, nursing, allied health, statisticians, data scientists, research coordinators, psychologists, pharmacists, health economists, and implementation scientists).
• Establish clear expectations around contributions as well as authorship and communication principles, and for larger projects consider a formal collaborative framework.
• Involve a diverse range of consumers (all ages with representation from minority groups and culturally and linguistically diverse backgrounds) in all stages from prioritization and research conceptualization through the research project and into implementation and translation.
• Incorporate early statistical expertise.
• Don’t neglect relationship factors such as fostering a respectful and psychologically safe environment with open communication to ensure a true research partnership.

In summary, a successful perioperative research team needs to incorporate not only clinicians, but also non-clinical multidisciplinary expertise, including a diverse range of consumers of all ages, to ensure the community truly has a voice. We all need to walk alongside one another, as equal partners, following the same vision, being respectful and willing to actively listen and learn from everyone. Such a team, comprising consumers, clinicians, non-clinical specialties, statisticians, data scientists, research coordinators/managers, health economists, and implementation scientists, will have a large talent pool with a range of expertise, skills and experience to drive the project successfully. The main challenge lies in creating a single, cohesive team that overcomes communication barriers, fosters a culture of open and safe communication, and thrives on the natural friction of different ideas and approaches. This will collectively shape our research into the best possible project, as all angles will be covered, and will help the rapid translation and implementation of findings into practice, reducing the amount of research waste.

## Declaration of competing interest

The authors declare no conflicts of interest.
